# Transcription Factors Sox8 and Sox10 Contribute with Different Importance to the Maintenance of Mature Oligodendrocytes

**DOI:** 10.3390/ijms25168754

**Published:** 2024-08-11

**Authors:** Lisa Mirja Jörg, Ursula Schlötzer-Schrehardt, Véronique Lefebvre, Elisabeth Sock, Michael Wegner

**Affiliations:** 1 Institut für Biochemie, Friedrich-Alexander-Universität Erlangen-Nürnberg, D91054 Erlangen, Germany; lisa.mirja.joerg@fau.de (L.M.J.); elisabeth.sock@fau.de (E.S.); 2 Department of Ophthalmology, Friedrich-Alexander-Universität Erlangen-Nürnberg, D91054 Erlangen, Germany; ursula.schloetzer-schrehardt@uk-erlangen.de; 3 Department of Surgery, Division of Orthopaedic Surgery, Children’s Hospital of Philadelphia, Philadelphia, PA 19104, USA; lefebvrev1@chop.edu

**Keywords:** glia, transcriptional control, myelin, Sox proteins, functional redundancy

## Abstract

Myelin-forming oligodendrocytes in the vertebrate nervous system co-express the transcription factor Sox10 and its paralog Sox8. While Sox10 plays crucial roles throughout all stages of oligodendrocyte development, including terminal differentiation, the loss of Sox8 results in only mild and transient perturbations. Here, we aimed to elucidate the roles and interrelationships of these transcription factors in fully differentiated oligodendrocytes and myelin maintenance in adults. For that purpose, we conducted targeted deletions of Sox10, Sox8, or both in the brains of two-month-old mice. Three weeks post-deletion, none of the resulting mouse mutants exhibited significant alterations in oligodendrocyte numbers, myelin sheath counts, myelin ultrastructure, or myelin protein levels in the corpus callosum, despite efficient gene inactivation. However, differences were observed in the myelin gene expression in mice with Sox10 or combined Sox8/Sox10 deletion. RNA-sequencing analysis on dissected corpus callosum confirmed substantial alterations in the oligodendrocyte expression profile in mice with combined deletion and more subtle changes in mice with Sox10 deletion alone. Notably, Sox8 deletion did not affect any aspects of the expression profile related to the differentiated state of oligodendrocytes or myelin integrity. These findings extend our understanding of the roles of Sox8 and Sox10 in oligodendrocytes into adulthood and have important implications for the functional relationship between the paralogs and the underlying molecular mechanisms.

## 1. Introduction

During ontogenetic development, oligodendrocytes arise via oligodendrocyte progenitor cells (OPCs) from neuroepithelial precursor cells in the ventricular zone of the vertebrate central nervous system (CNS) [1,2]. As part of their differentiation, oligodendrocytes form myelin sheaths around axonal segments and thereby allow saltatory conduction and fast information processing [3]. As a consequence, they are important components of neural circuits in the CNS [4].

Over the years, several transcription factors have been identified that are part of the oligodendroglial regulatory network and influence oligodendrocyte development and myelination [5,6,7]. This includes the HMG-box transcription factor Sox10 and its close relative Sox8 [5,8]. Both proteins are expressed throughout lineage development from OPC to fully mature oligodendrocytes [5,9]. Although Sox10 has a strong influence during all stages of oligodendroglial development [5], Sox8 loss causes only a mild delay of differentiation in early postnatal oligodendrocytes [9]. The most prominent role of Sox8 in oligodendroglial cells has been established in mouse mutants with a constitutive Sox8 deficiency and a *Mog::iCre*-induced Sox10 loss [10]. Oligodendroglial cells in these mice lack Sox8 at all times and additionally lose Sox10 during the late stages of the myelination process. While still normal at early phases of myelination, the expression of myelin genes was heavily disturbed during the late phases of myelination in these mice. In contrast, mice that carried only one or the other mutation did not exhibit any conspicuous abnormalities in myelin gene expression. From these analyses, it was concluded that the remaining presence of either Sox8 or Sox10 is sufficient to complete the already induced oligodendroglial differentiation and maintain the differentiated state [10]. Due to the ability of Sox8 and Sox10 to compensate the loss of the other, both factors appeared functionally redundant in myelin-forming oligodendrocytes.

Here, we extended our analysis to adult mice and revisited the relationship of Sox8 and Sox10 by inactivating both factors in a tamoxifen-dependent manner at 2 months using a *Plp1::CreERT2* Bac transgene [11]. Previous studies on 4-month-old *Sox10^fl/fl^ Plp1::CreERT2* mice (henceforth referred to as Sox10cko) have already shown that such mice develop a severe peripheral neuropathy after approximately a month of tamoxifen treatment [12]. This is due to the additional activity of the Cre transgene in peripheral glia and the known role of Sox10 in Schwann cells [11,13,14]. Compared to this previous study, we included Sox8 in the analysis. Using both *Sox10^fl^* and *Sox8^fl^* alleles, we deleted both factors simultaneously and after oligodendrocyte differentiation was completed so that all determined alterations exclusively concerned maintenance issues. By the inclusion of expression profiling, our goal was to gain novel insights into the relationship between Sox8 and Sox10 and the mechanisms underlying their function.

## 2. Results

### 2.1. Ablation of Sox8 and Sox10 in Adult Oligodendrocytes is Efficient

To study the consequences of Sox8 and Sox10 inactivation in adult oligodendrocytes, we combined floxed alleles for *Sox8* and *Sox10*, either alone or in combination, with a *Plp1::CreERT2* transgene and treated these animals at two months of age (day 60) for five consecutive days with tamoxifen. Under this regimen, severe dysfunctions of the peripheral nervous system became evident after the third week in Sox10cko and *Sox8^fl/fl^ Sox10^fl/fl^ Plp1::CreERT2* (from now on referred to as dcko) mice. Consequently, analysis was performed at 21 days post injection (dpi).

**Figure 1:**
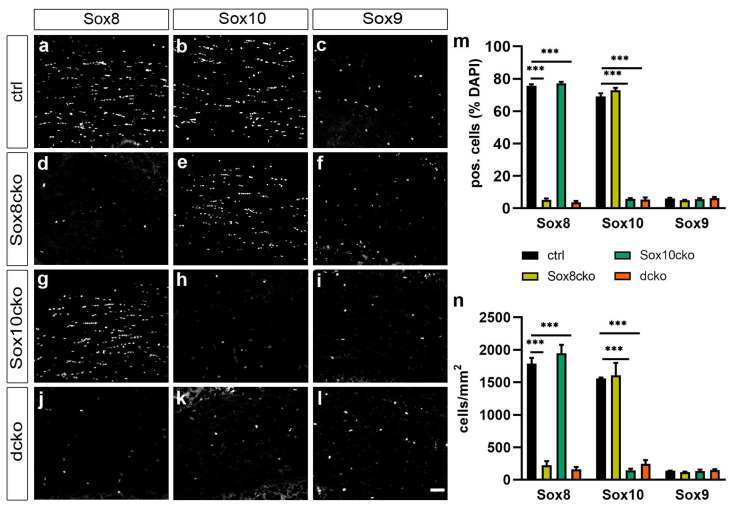
Efficient gene inactivation in mutant mice. (**a**–**l**) Co-immunohistochemical stainings of the corpus callosum from the control mice (ctrl, (**a**–**c**)), Sox8 mutant mice (Sox8cko, (**d**–**f**)), Sox10 mutant mice (Sox10cko, (**g**–**i**)), and Sox8/Sox10 double-mutant mice (dcko, (**j**–**l**)) at 21 dpi, with antibodies directed against Sox8 (**a**,**d**,**g**,**j**), Sox10 (**b**,**e**,**h**,**k**), and Sox9 (**c**,**f**,**i**,**l**). Scale bar: 50 µm. Microscope enlargement: 200×. (**m**) Quantification of the percentage of Sox8-, Sox10-, or Sox9-expressing cells among all cells in the corpus callosum (identified by DAPI stain) in the various mouse lines. (**n**) The absolute numbers of cells expressing Sox8, Sox10, or Sox9 per mm^2^ in the corpus callosum of the various mouse lines. Bar graphs show the mean ± standard error of the mean (n = 4). The statistical significance relative to the controls was determined for each Sox protein by Student’s *t*-test (***, *p* ≤ 0.001).

For the analysis, we focused on the corpus callosum as a representative white matter tract. By staining the forebrain sections for Sox8, Sox10, and the third paralog of subgroup E of the Sox family Sox9, we were able to show that 75.8 ± 0.6% of all cells in the corpus callosum exhibited immunoreactivity for Sox8 in the control mice (Figure 1a,m). The number of cells with Sox10 expression was comparable and reached 71.2 ± 1.4% (Figure 1b,m). In contrast, only 6.1 ± 0.4% of the cells in the corpus callosum were stained for Sox9 (Figure 1c,m).

When the same analysis was carried out in *Sox8^fl/fl^ Plp1-CreERT2* (referred to as Sox8cko) mice, similar values were obtained for the cells expressing Sox10 (72.7 ± 1.0%) or Sox9 (5.4 ± 0.3%), while the proportion of Sox8-expressing cells decreased to 5.2 ± 0.8% (Figure 1d–f,m). Similarly, the number of cells in Sox10cko mice with Sox8 (77.3 ± 0.7%) or Sox9 (5.6 ± 0.6%) expression were comparable to those in the corpus callosum of the control mice (Figure 1g,i,m). In these mice, the number of Sox10-expressing cells selectively dropped to 5.9 ± 0.4% (Figure 1h,m). In the corpus callosum of the dcko mice, both the number of Sox8-expressing and Sox10-expressing cells were similarly reduced (3.7 ± 0.7% and 5.4 ± 1.2%, respectively), notably without an increase in the number of cells expressing Sox9 (Figure 1j–m). In line with these findings, the absolute numbers of Sox8- or Sox10-expressing cells were likewise reduced in the corpus callosum of the corresponding mouse mutants (Figure 1n). The Sox8 and Sox10 deletion in oligodendroglial cells reached approximately 90–95% in the various mutants.

### 2.2. The Number and State of Oligodendroglial Cells Remain Unchanged upon Loss of Sox8 and Sox10

**Figure 2:**
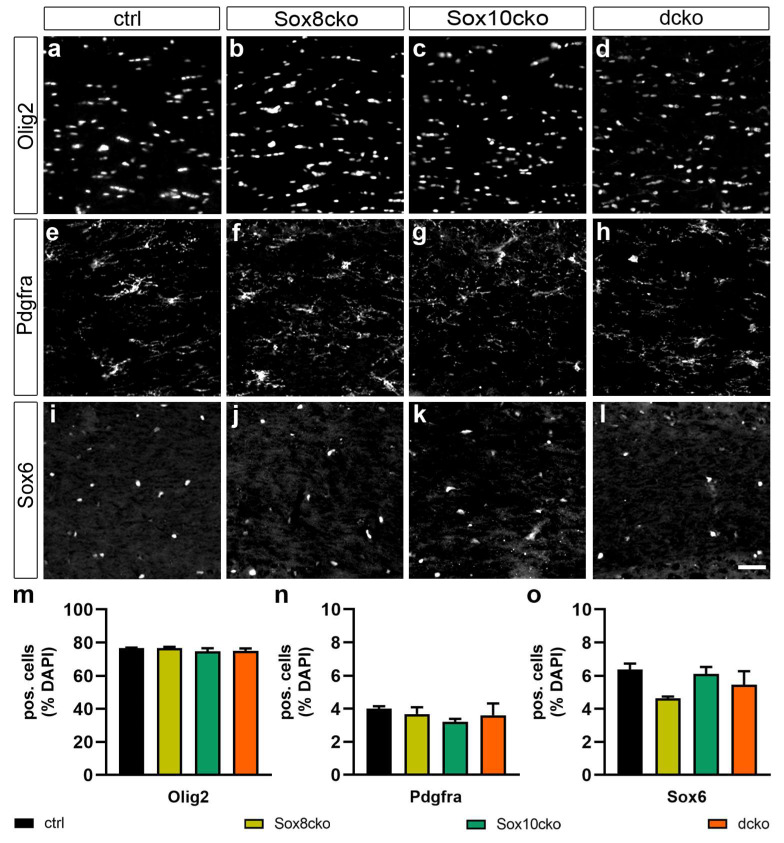
Unchanged oligodendroglial cell numbers in mutant mice with Sox8 and Sox10 deletions. (**a**–**l**) Immunohistochemical stainings of the corpus callosum from the control mice (ctrl, (**a**,**e**,**i**)), Sox8 mutant mice (Sox8cko, (**b**,**f**,**j**)), Sox10 mutant mice (Sox10cko, (**c**,**g**,**k**)), and Sox8/Sox10 double-mutant mice (dcko, (**d**,**h**,**l**)) at 21 dpi, with antibodies directed against Olig2 (**a**–**d**), Pdgfra (**e**–**h**), and Sox6 (**i**–**l**). Scale bar: 50 µm. Microscope enlargement: 200×. (**m**–**o**) Quantification of the percentage of cells expressing Olig2 (**m**), Pdgfra (**n**), or Sox6 (**o**) among all the cells in the corpus callosum (identified by DAPI stain) of the various mouse lines. Bar graphs show the mean ± standard error of the mean (n = 4). No statistical significance was determined for any of the mutants or markers by Student’s *t*-test.

To characterize the consequences of the loss of Sox8 and Sox10, we first quantified the number of Olig2-positive cells in the corpus callosum of the various mouse lines at 21 dpi. As evident from the representative pictures (Figure 2a–d) and corresponding quantifications (Figure 2m), Olig2-expressing cells were comparable in number among all mouse lines and made up 74.6–76.6% of all cells in the corpus callosum.

As Olig2 does not distinguish between adult OPCs and oligodendrocytes, we next performed stainings and quantifications for Pdgfra, a protein that selectively labels the OPC fraction. Using this marker, we identified 3.2–4.0% of all cells in the corpus callosum as OPCs but failed to detect significant differences in the cell numbers between controls and the various Sox mutant lines (Figure 2e–h,n). This result was confirmed by staining for Sox6 (Figure 2i–l,o), whose expression in the corpus callosum of adult mice was effectively restricted to OPCs [5]. Neither the total number of oligodendroglial cells nor the population of OPCs was substantially affected after the individual or combined ablation of Sox8 and Sox10.

### 2.3. Changes in Myelin Are Subtle after the Deletion of Sox8 and Sox10 in Adult Oligodendrocytes

**Figure 3:**
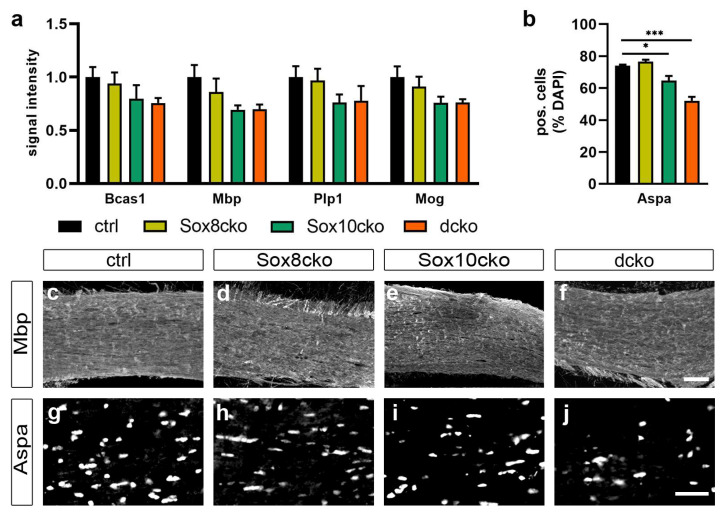
Mild reductions in the myelin protein amounts in mutant mice with Sox8 and Sox10 deletions. (**a**) Quantification of signal intensities for Bcas1, Mbp, Plp1, and Mog following immunohistochemical staining of the corpus callosum of the control (ctrl), Sox8 mutant (Sox8cko), Sox10 mutant (Sox10cko), and Sox8/Sox10 double-mutant (dcko) mouse lines at 21 dpi. Bar graphs show the mean ± standard error of the mean (n = 4) for each genotype with intensities for the control animals set to 1. (**b**) Quantification of the percentage of the Aspa-expressing cells among all cells (identified by DAPI stain) in the corpus callosum of the various mouse lines. Statistical significance relative to the controls was determined for each marker by Student’s *t*-test (*, *p* ≤ 0.05; ***, *p* ≤ 0.001). (**c**–**j**) Exemplary immunohistochemical stainings of the corpus callosum from the control (**c**,**g**), Sox8cko (**d**,**h**), Sox10cko (**e**,**i**), and dcko (**f**,**j**) mice with antibodies directed against Mbp (**c**–**f**) and Aspa (**g**–**j**). Scale bar: 50 µm. Microscope enlargement: 200×.

The Bcas1 (breast carcinoma amplified sequence 1) protein has been reported to be preferentially expressed in newly generated oligodendrocytes in demyelinating lesions [15]. Following immunohistochemical staining, the intensity of the Bcas1 signal was comparable between the corpus callosum of the control and Sox8cko mice, and exhibited a mild decrease in the Sox10cko and dcko mice (signal intensities of 0.80 and 0.76 relative to 0.9 and 1.0 in the Sox8cko and control mice; Figure 3a). However, this decrease did not reach statistical significance (Figure 3a). Similar results were obtained in immunohistochemical stainings for three major myelin proteins, i.e. the myelin basic protein (Mbp), the proteolipid protein 1 (Plp1), and the myelin oligodendrocyte protein (Mog). Only Sox10cko and dcko mice showed a mild reduction in the Mbp, Plp1, and Mog signal intensities (Figure 3a,c–f). In the case of Plp1, for instance, relative signal intensities were reduced by 22–24% in the Sox10cko and dcko mice, but only by 3% in the Sox8cko mice relative to the controls. Again, this reduction did not reach statistical significance for any of the markers. In contrast, reductions in the number of aspartoacylase (Aspa) expressing oligodendrocytes in the corpus callosum of the Sox10cko and dcko mice were statistically significant (Figure 3b,g–j). They were more pronounced in the dcko mice (52 ± 2% of all cells compared to 74.0 ± 1% in the controls) than in the Sox10cko mice (64 ± 7% compared to 74.0 ± 1% in the controls).

**Figure 4:**
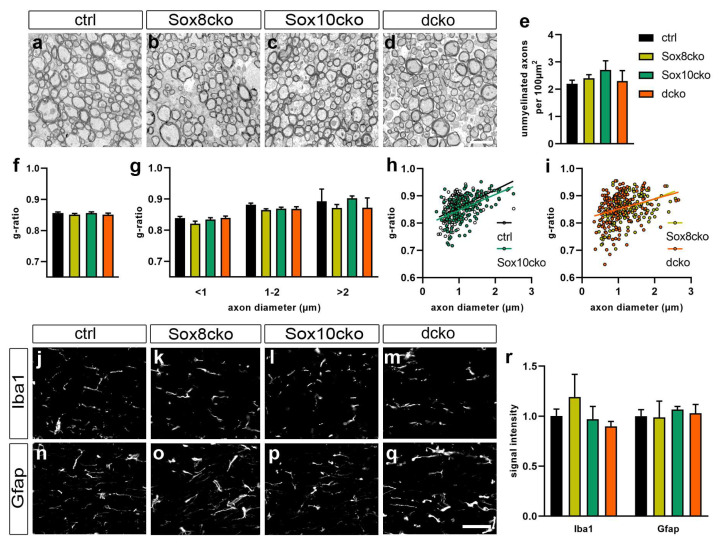
Unaffected myelin sheaths in mutant mice with Sox8 and Sox10 deletions. (**a**–**d**) Electron microscopic pictures of the sagittally cut corpus callosum from the control mice (ctrl, (**a**)), Sox8 mutant mice (Sox8cko, (**b**)), Sox10 mutant mice (Sox10cko, (**c**)), and Sox8/Sox10 double-mutant mice (dcko, (**d**)) at 21 dpi. Electron microscope enlargement: 3600×. (**e**) Quantification of the absolute number of unmyelinated axons per 100 µm^2^ of sagittally cut corpus callosum in the various mouse mutants. (**f**–**i**) Representation of the g-ratios in the corpus callosum of the controls and the various mouse mutants, as determined over all axons (**f**) after binning of axons according to diameter size (**g**) or presented for single axons in a scatter plot (**h**,**i**). (**j**–**r**) Immunohistochemical stainings of the corpus callosum from the control (**j**,**n**), Sox8cko (**k**,**o**), Sox10cko (**l**,**p**), and dcko (**m**,**q**) mice at 21 dpi, with antibodies directed against Iba1 (**j**–**m**) and Gfap (**n**–**q**), as well as the corresponding quantification of signal intensities (**r**). Bar graphs show the mean ± standard error of the mean (n = 4) for each genotype, with intensities for the control animals set to 1. No statistical significance relative to the controls was determined for any of the parameters or markers by Student’s *t*-test. Scale bar: 50 µm. Microscope enlargement: 200×.

In line with persistently high levels of myelin proteins, electron microscopy on the corpus callosum did not reveal any obvious structural alterations in the myelin sheaths of the Sox8cko, Sox10cko, or dcko mice when compared to the controls (Figure 4a–d). There was no increased occurrence of abnormal myelin structures or myelin debris. The number of unmyelinated axons was not increased in any of the genotypes and ranged from 2.7 to 2.2 unmyelinated axons per 100 µm^2^ (Figure 4e). Additionally, the g-ratio as a measure of myelin thickness was comparable between all genotypes (0.85–0.86 in all genotypes), not only when averaged over all axons (Figure 4f), but also when separately determined for axons of small, middle, or large diameter sizes (Figure 4g–i). Even 21 days after tamoxifen-induced gene deletion, the levels of the myelin proteins remained sufficient to maintain intact and morphologically inconspicuous myelin sheaths in all mutant genotypes, including dcko mice.

Histology and immunohistochemical staining for Iba1 showed no signs of microglial activation or increased immune cell infiltration (Figure 4j–m,r). Additionally, immunohistochemical staining for Gfap was comparable among all genotypes, with signal intensities varying no more than 7% among all genotypes. There were no signs of astroglial activation or astrogliosis in any of the mutant genotypes (Figure 4n–r).

### 2.4. Gene Expression Changes Substantially in the Adult Oligodendrocytes of Sox10cko and Dcko Mice

**Figure 5:**
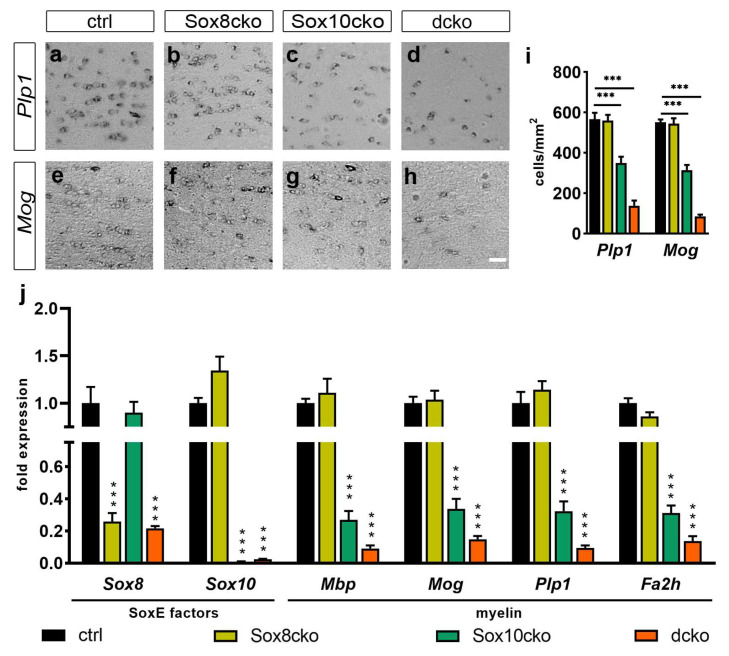
Altered expression of the myelin genes in mutant mice with Sox8 and Sox10 deletions. (**a**–**i**) In situ hybridization of the corpus callosum from the control (ctrl, **a**,**e**), Sox8 mutant (Sox8cko, **b**,**f**), Sox10 mutant (Sox10cko, **c**,**g**), and Sox8/Sox10 double-mutant (dcko, **d**,**h**) mice at 21 dpi with antisense probes directed against *Plp1* (**a**–**d**) and *Mog* (**e**–**h**), as well as corresponding quantifications (**i**). The bar graph shows the absolute numbers of the transcript-positive cells per mm^2^ in the corpus callosum of the various mouse lines as the mean ± standard error of the mean (n = 4) for each genotype. Scale bar: 50 µm. Microscope enlargement: 200×. (**j**) Expression of the *Sox8*, *Sox10*, *Mbp*, *Mog*, *Plp1*, and *Fa2h* in the corpus callosum from the control and mutant mice, as determined by quantitative RT-PCR. Values represent the mean relative expression levels ± standard error of the mean (n = 4 per genotype), with transcript levels of each marker set to 1 in the controls. Statistical significance relative to the controls was determined for each marker by Student’s *t*-test (***, *p* ≤ 0.001).

Considering the fact that myelin proteins rank among the most stable proteins in the mammalian CNS, we reasoned that much of the myelin proteins present at 21 dpi were generated before tamoxifen-dependent gene ablation and therefore mask the effects of gene loss. To bypass this complication, we focused on myelin gene transcripts as a proxy for the newly generated myelin proteins. Interestingly, in situ hybridizations for *Plp1* and *Mog* transcripts revealed significant reductions in the number of transcript-positive cells in the corpus callosum of the Sox10cko mice, and there were even stronger reductions in the dcko mice (Figure 5a,c,d,e,g–i). In contrast, the number of *Plp1*-positive and *Mog*-positive cells was comparable to controls in the corpus callosum of the Sox8cko mice (Figure 5a,b,e,f,i). A reduction in *Mbp, Plp1, Mog*, and *Fa2h* transcripts was also detected for the Sox10cko and dcko mice when cDNA was prepared from total corpus callosum RNA and analyzed by qRT-PCR (Figure 5j). By the same approach, we were also able to confirm the reduction in *Sox8* and *Sox10* transcripts in the mutants where the genes were targeted by Cre activity.

Based on the fact that the consequences of gene deletions were much more readily detectable on the transcript level, we decided to use corpus callosum RNA for a more global expression profiling exercise. In this approach, we compared expression patterns in the control mice to those in the Sox8cko, Sox10cko, or dcko mice by RNA sequencing. Analysis of the sequencing data revealed that the transcript levels for *Sox8* and *Sox10* were approximately equivalent in the adult control oligodendrocytes, with 147.6 ± 13.1 specific transcripts per million transcripts (TPM) for Sox8 and 198.5 ± 15.1 TPM for Sox10. RNA sequencing also confirmed the efficient gene deletion in the various mouse lines. *Sox8* transcript levels were reduced to 23.2 ± 4.2 TPM in mice with oligodendrocyte-specific Sox8 ablation. A comparable reduction to 15.3 ± 1.6 TPM was observed for the *Sox10* transcripts in the mice with Sox10 deletion. Intriguingly, *Sox9* transcript levels remained around 28–52 TPM in all samples, independent of whether Sox8, Sox10, or both were deleted.

**Figure 6:**
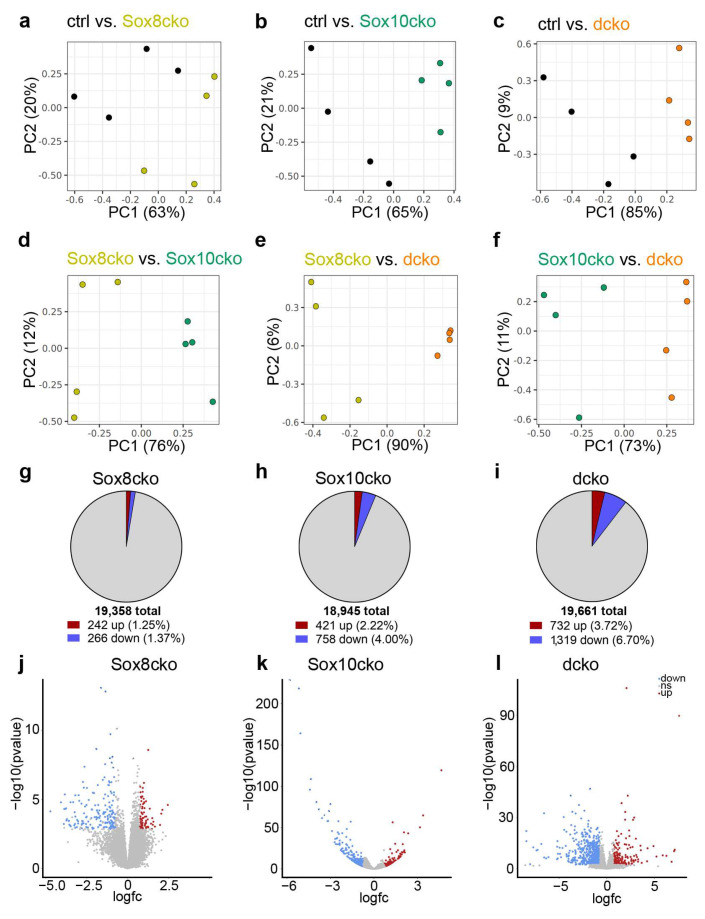
Changed expression profiles in the corpus callosum of mice with oligodendrocyte-specific deletions of Sox8 and Sox10. (**a**–**f**) PCA plots showing pairwise comparisons of the results from the control (ctrl), Sox8 mutant (Sox8cko), Sox10 mutant (Sox10cko), and Sox8/Sox10 double-mutant (dcko) mice obtained by RNA sequencing of corpus callosum samples. (**g**–**i**) Pie charts summarizing the number of upregulated (red) or downregulated (blue) differentially expressed genes (DEGs, as defined by a log2-fold change of ≥±0.75, *p*-value of ≤0.05, and a mean base count of >20 gene-specific transcripts per million transcripts) in the corpus callosum of the Sox8cko (**g**), Sox10cko, (**h**) and dcko mice. (**j**–**l**) Graphical representation of the DEGs in Sox8cko (**j**), Sox10cko (**k**), and dcko (**l**) mice according to the *p*-value and log2-fold change (logfc) in Volcano plots.

As evident from the principal component analysis (PCA) plots, there was a clear difference in the expression profiles between the control mice and the Sox10cko or dcko mice, whereas the difference between the controls and the Sox8cko mice was much less pronounced (Figure 6a–c). Additionally, the expression profiles of the mice with individual Sox8 or Sox10 deletions were distinct from each other, as well as from the dcko mice (Figure 6d–f).

Quantitative analysis of the differentially expressed genes (DEGs) also showed that the overall changes in the expression profile were considerably smaller for Sox8cko mice than for the other mutants. While ~10% of all genes expressed in the corpus callosum of the dcko mice qualified as DEGs (as defined by a log2-fold change of ≥±0.75, *p*-value of ≤0.05, and a mean base count of >20 TPM), this number dropped to ~6% for the Sox10cko mice and a mere ~3% for the Sox8cko mice (Figure 6g–i). Interestingly, the downregulated genes outnumbered the upregulated genes in both Sox10cko and dcko mice. In contrast, the number of upregulated genes was comparable to the number of downregulated genes among the DEGs identified in the Sox8cko mice.

DEGs with very high probability (−log10 *p*-value ≥ 20) were exclusively observed in the samples from the Sox10cko and dcko mice, and they were not present in the samples from the Sox8cko mice, as evident from the Volcano plots (Figure 6j–l). Notably, the highest rates of upregulation or downregulation (log2fold change of ≥±5) were restricted to DEGs from the dcko mice.

**Figure 7:**
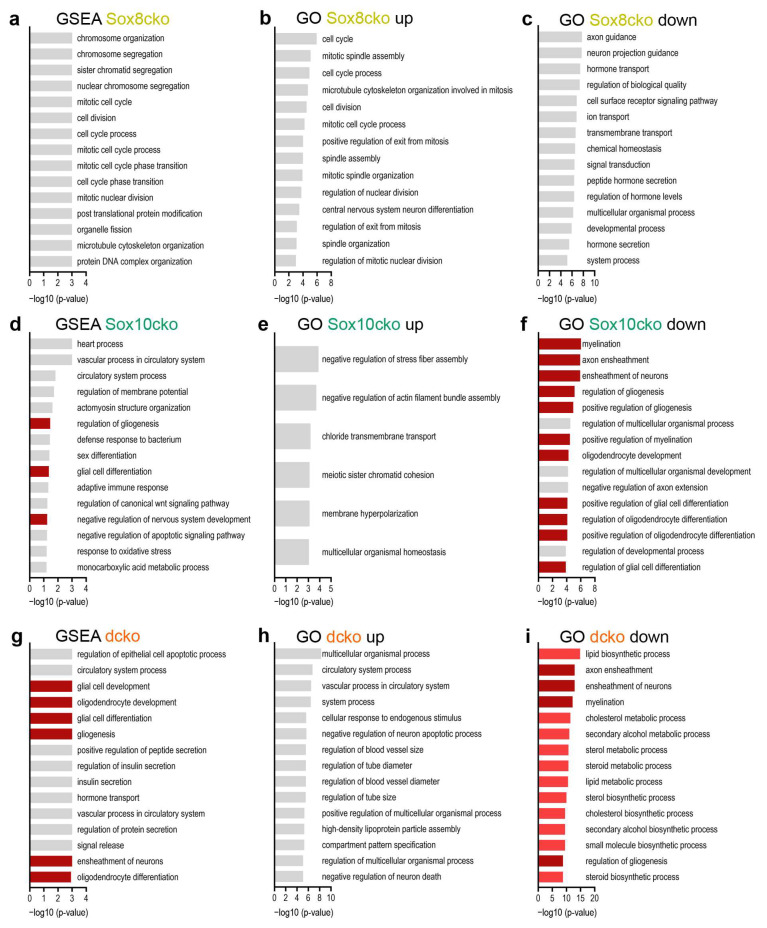
Altered cellular processes in the corpus callosum of mice with oligodendrocyte-specific deletion of Sox8 and Sox10. (**a**–**i**) Gene set enrichment analyses (GSEA; (**a**,**d**,**g**)) and gene ontology (GO) studies on the upregulated (**b**,**e**,**h**) and downregulated (**c**,**f**,**i**) differentially expressed genes (as defined by a log2-fold change of ≥±0.75, *p*-value of ≤0.05, and a mean base count of >20 gene-specific transcripts per million transcripts) according to the RNA-sequencing data from the corpus callosum of the Sox8 mutant (Sox8cko, (**a**–**c**)), Sox10 mutant (Sox10cko, (**d**–**f**)), and Sox8/Sox10 double-mutant (dcko, (**g**–**i**)) mice. The top 15 terms (if present) according to adjusted *p*-values are listed. Terms related to glia are labeled in dark red, those related to lipid and cholesterol metabolism are in light red; all others are in gray.

Gene set enrichment analysis (GSEA) further revealed that the loss of Sox8 primarily affected the general organization of chromatin and microtubules, as well as the DNA and cell cycle characteristics (Figure 7a). Similar terms were annotated when gene ontology (GO) analysis was performed on the upregulated genes (Figure 7b). For the GO analysis of the downregulated genes, terms were related to axon guidance, ion transport, or signal transduction (Figure 7c). Intriguingly, neither GSEA nor GO analysis revealed a specific link to glial cells.

In contrast, analogous GSEA on the Sox10cko mice detected alterations in gene sets associated with regulation of gliogenesis and glial cell differentiation (Figure 7d). There was again no clear link to the glia-related processes among the few terms that were retrieved by GO analysis of the genes upregulated in the Sox10cko samples (Figure 7e). In contrast, when the genes downregulated in Sox10cko mice underwent GO analysis, terms related to myelination, axon ensheathment, and oligodendrocyte development were significantly enriched in addition to a regulation of gliogenesis and glial cell differentiation (Figure 7f). Not surprisingly, the link to glial biology, oligodendrocytes, and ensheathment was even more prominent in GSEA on dcko mice (see Figure 7g, also note the higher *p*-values than in Figure 7d). In the GO analysis, these terms segregated exclusively with the downregulated genes (compare Figure 7h,i). For the downregulated genes, additional terms appeared in the GO analysis that were related to lipid biosynthesis and cholesterol metabolism and thus highly relevant to myelination and myelin maintenance (Figure 7i). In contrast, the GO analysis on the upregulated genes in the dcko mice did not yield terms related to glial cells or myelin but revealed a link to the altered regulation of (neuronal) apoptosis (Figure 7h). Terms related to astrocytes, microglia, inflammation, or other immune-related terms were not identified in the GSEA or GO analysis for any of the mouse mutants.

**Figure 8:**
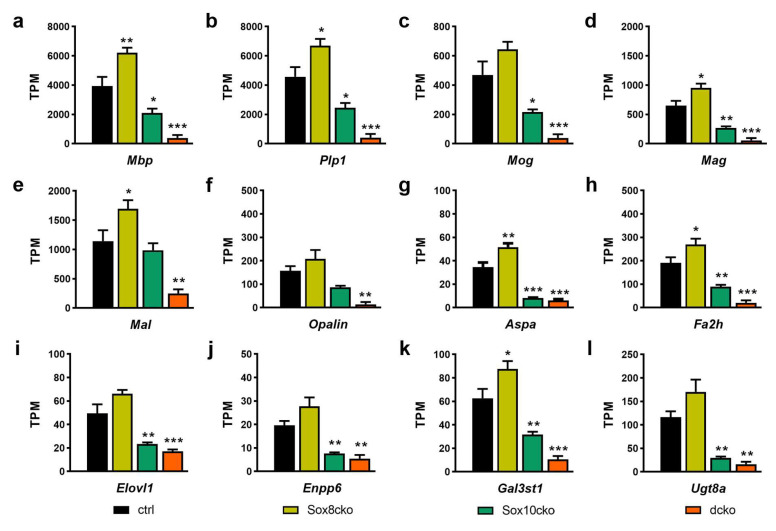
Altered expression of select genes in the corpus callosum of the Sox8 mutant (Sox8cko), Sox10 mutant (Sox10cko), and Sox8/Sox10 double-mutant (dcko) mice. (**a**–**l**) Expression levels of *Mbp* (**a**), *Plp1* (**b**), *Mog* (**c**), *Mag* (**d**), *Mal* (**e**), *Opalin* (**f**), *Aspa* (**g**), *Fa2h* (**h**), *Elovl1* (**i**), *Enpp6* (**j**), *Gal3st1* (**k**), and *Ugt8a* (**l**) in the control, Sox8cko, Sox10cko, and dcko mice according to RNA-sequencing data and are represented as gene-specific transcript per million transcripts (TPM). Statistical significance relative to the controls was determined for each marker by Student’s *t*-test (*, *p* ≤ 0.05; **, *p* ≤ 0.01; and ***, *p* ≤ 0.001).

When analyzing the RNA-sequencing results for the expression levels of key genes related to myelin or lipid metabolism, we detected a substantial downregulation in the samples from the dcko mice (Figure 8a–l). In the RNA from the corpus callosum of the Sox10cko mice, the expression of a few of these key genes remained unaffected (e.g., *Mal*, see Figure 8e), while the expression of most was lower than in the control samples. The transcript amounts for the genes with reduced expression in the Sox10cko mice were usually closer to those in the controls than the dcko mice. However, for certain genes, the reductions in the Sox10cko mice were fairly close to those observed in the dcko mice (e.g., *Aspa*, *Enpp6*, or *Ugt8a*; see Figure 8g,e,l). Strikingly, none of the genes related to myelin or lipid metabolism exhibited reduced expression levels in the Sox8cko mice. Instead, some genes even showed increased expression.

**Figure 9:**
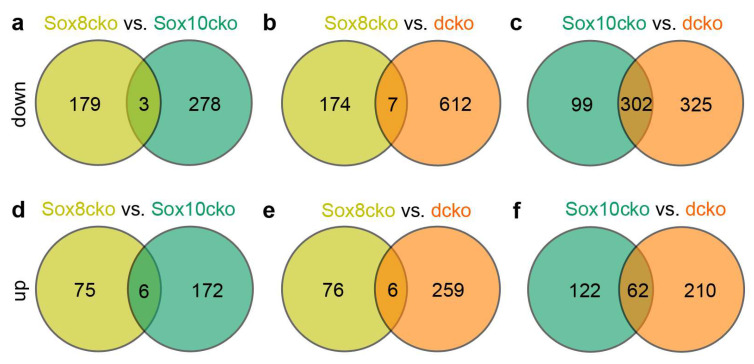
Comparison of the expression changes in mice with oligodendrocyte-specific deletion of Sox8 and Sox10. (**a**–**f**) Venn diagrams showing the overlap of upregulated (**a**–**c**) and downregulated (**d**–**f**) genes between the Sox8 mutant (Sox8cko) and Sox10 mutant (Sox10cko) mice (**a**,**b**); Sox8cko and Sox8/Sox10 double-mutant (dcko) mice (**c**,**d**); or the Sox10cko and dcko (**e**,**f**) mice. Jointly deregulated genes were defined as genes that are similarly up or downregulated in both mutant mice with a *p*-value ≤ 0.05 and achieving a log2fold deregulation of ≥±0.75 in at least one mutant.

A comparison of the upregulated or downregulated DEGs between Sox8cko on the one side and Sox10cko or dcko on the other side further revealed that there was virtually no overlap (i.e., ≤1% of the sum of DEGs in both compared mouse mutants) (Figure 9a,b,d,e). In contrast, the overlap between Sox10cko and dcko amounted to 16% of all DEGs for the upregulated genes and 42% for the downregulated genes (Figure 9c,f). The strong alterations in the expression of the genes relevant for the oligodendrocytes and myelin in the dcko mice were partially already present at moderate levels in the Sox10cko mice but not in the Sox8cko mice.

## 3. Discussion

Previous studies had shown that Sox8 is co-expressed with Sox10 at all times of oligodendroglial development, but has only limited influence on lineage progression, the induction of terminal differentiation, and the myelination process despite its close structural resemblance to Sox10 [9]. Sox8 appears to become more relevant only at late stages of the developmental myelination process, as indicated by the fact that the combined deletion of both Sox8 and Sox10 is necessary to severely affect late-stage myelination [10]. In the current study, we compare the role of these two related Sox transcription factors on myelin maintenance in the forebrain of two-month-old mice.

By using floxed *Sox8* and *Sox10* alleles in combination with a tamoxifen-inducible *Plp1-CreERT2*, we inactivated both genes in the myelin-forming oligodendrocytes of the CNS. In the corpus callosum, oligodendroglial cells make up approximately two thirds to three quarters of all cells [3]. From our immunohistochemical quantifications and RNA-seq results, we can conclude that the deletion of both genes in oligodendroglial cells was similarly efficient and amounted to approximately 90–95% in the various mutants. The remaining Sox8- and Sox10-expressing cells likely correspond to adult OPCs as activity of the Cre recombinase is restricted to the oligodendrocyte stage and is not present in OPCs.

We also analyzed the expression of the closely related Sox9 in the corpus callosum and noted that the number of Sox9-expressing cells was substantially lower than the number of oligodendroglial cells. This agrees with previous reports that Sox9 occurs in oligodendroglial cells during developmental myelination but not in the adult brain, where its occurrence is restricted to astrocytes [16,17]. Interestingly, neither the number of Sox9-expressing cells nor the overall level of *Sox9* transcripts changed in any of the mutants. This suggests that there is no compensatory upregulation of Sox9 in oligodendrocytes following the deletion of Sox8, Sox10, or both. This is an important observation because such an upregulation could mask the gene-deletion effects.

Tamoxifen-dependent induction of the *Plp1-CreERT2* transgene also caused efficient gene ablation in the peripheral nervous system. The loss of Sox10 in particular led to severe peripheral problems that also limited our window of analysis in the central nervous system to three weeks after tamoxifen injection. At 21 dpi, the myelin ultrastructure in the corpus callosum appeared intact in all mouse mutants, independent of whether Sox8, Sox10, or both were inactivated. Reductions in the amounts of myelin proteins in the corpus callosum were mild and exclusively observed in the Sox10cko and dcko mice. Furthermore, the three myelin proteins analyzed were still present at very high levels such that the observed reductions failed to reach statistical significance. A more pronounced decrease was detected in the Sox10cko and dcko mice for Aspa, a protein that is restricted to mature oligodendrocytes. The findings suggest that a fraction of the oligodendrocytes no longer expressed the Aspa protein.

We believe that our inability to detect dramatic changes in the myelin structure or severe reductions in myelin proteins was mainly due to the known stability of myelin and its components in the adult brain [18,19]. That myelin was still sufficiently stable at the time of analysis was further supported by the results from the immunohistochemical stainings of Iba1, Gfap, or Sox9. These stainings were all inconspicuous and did not exhibit increased signal intensities or numbers of marker-positive cells, which would be indicative of inflammatory responses to myelin changes, such as microgliosis or astrogliosis [20].

In contrast to the mild changes in the amounts of myelin proteins and in the myelin structure, more substantial alterations were detected in the transcript levels of the myelin-related genes in the corpus callosum of the Sox10cko and dcko mice. In particular, the RNA-sequencing results suggest that there was a significant difference in the extent to which the expression of the myelin-related genes was reduced in the Sox10cko and dcko mice. The pronounced reductions in the dcko mice allowed the prediction that myelin maintenance would be affected at later times. The myelin-related changes in the Sox10cko mice were much milder such that it is unclear if and when such changes would lead to an overt demyelinating phenotype in the mouse. The clear-cut difference in the observed changes between the dcko and Sox10cko mice confirms the joint requirement of Sox8 and Sox10 for myelin maintenance. Similar results have been described in a previous study for late-stage myelination during the early postnatal period [10]. Our present study extends the previous findings to mature oligodendrocytes of the adult brain.

Due to the substantial alterations observed in the dcko mice, we had expected to find at least mildly disturbed expressions of myelin-related genes in both the Sox8cko and Sox10cko mice. However, the Sox8cko mice did not exhibit any alterations in the expression of genes related to myelin or lipid metabolism. In fact, the overall changes in the expression profile were very subtle when compared to the controls. Furthermore, there was hardly any overlap between the genes differentially expressed in the Sox8cko mice on the one hand and Sox10cko or dcko mice on the other. Therefore, it was not immediately obvious how Sox8 contributes to myelin maintenance in adult oligodendrocytes.

Around birth, Sox8 is expressed at substantially lower levels in oligodendroglial cells than Sox10, and this difference in expression may explain the lesser impact of Sox8 on oligodendrocyte differentiation and the initiation of myelination [9]. For adult oligodendrocytes, however, our RNA-sequencing data show that transcript levels for *Sox8* and *Sox10* are comparable such that the differences in amounts are an unlikely explanation for the different influence on myelin gene expression at this stage. While we are aware that there is no strict correlation between transcript and protein levels, the signal intensity in immunohistochemical stainings also indicates that Sox8 amounts in adult oligodendrocytes are substantial.

With comparable levels of Sox8 and Sox10 in adult oligodendrocytes, it is not immediately obvious why strong alterations in the expression of myelin-related genes are observed only in the absence of both closely related Sox proteins when Sox8 deletion has no influence on its own. It seems that the loss of Sox8 remains without consequence in the context of myelin-related gene expression as long as Sox10 is still present. Once Sox10 is lost, however, Sox8 seems to be able to take over at least part of the functions normally performed by Sox10. This suggests that differences exist between the closely related paralogous proteins.

While we cannot exclude that Sox8 and Sox10 recognize different target sites and have distinct functions, we favor a model in which the two proteins are functionally similar and generally bind to the same target sites. Assuming the presence of comparable amounts of Sox8 and Sox10 protein in mature oligodendrocytes and a total amount of combined Sox8 and Sox10 protein that exceeds the demand for myelin maintenance and the number of binding sites in target genes, Sox10 could simply be more efficient than Sox8 in exerting these common functions and thus outcompetes Sox8. As the domains involved in DNA-binding (i.e., the high-mobility-group domain and the dimerization domain) are virtually identical between Sox8 and Sox10 [21], the two proteins are likely to have a similar affinity for the same target sites. However, the two proteins differ substantially in their transactivation domains. As a consequence, Sox8 may be less efficient than Sox10 in integrating into active transcriptional complexes, interacting with partnering transcription factors, or recruiting chromatin modifiers or remodelers that are required for myelin maintenance [22,23]. Under normal conditions in mature oligodendrocytes, both Sox8 and Sox10 may bind to their target sites and the overall target gene activation will be the average of the weaker activation by Sox8 and the stronger activation by Sox10. When Sox8 is lost, target gene activation may not be affected or even slightly increased because Sox10 no longer competes with Sox8. When Sox10 is lost, the remaining amounts of the less efficient Sox8 will be insufficient to fully maintain normal expression levels. Dramatic reductions will, however, require the loss of both proteins.

In several other cell types, Sox8 is co-expressed with Sox9 [24,25,26]. As already mentioned, Sox9 is highly related to Sox8 and Sox10, and it represents the third paralog of group E of the Sox family of transcription factors. Co-expression occurs, for instance, in sex determination, Sertoli cells, and chondrocyte development. In these processes, Sox9 is usually a driving force [27], whereas the effects of Sox8 are often more subtle [24,25,26]. Therefore, the relationship between Sox8 and Sox9 in these tissues is reminiscent of the one between Sox8 and Sox10 in oligodendrocytes. Similar patterns of co-expression and partial functional redundancy have also been observed for paralogous Sox proteins from other groups, including group B1, group C, and group D [28,29,30,31]. In all these cases, it will be interesting to see whether there are similarities in the underlying molecular mechanisms that determine the partial functional redundancy.

## 4. Materials and Methods

### 4.1. Mice

The mice with inducible knockouts of *Sox8*, *Sox10*, or both in adult oligodendrocytes were generated by a combination of *loxP* sites containing (floxed) *Sox8* (*Sox8^flox^*) [24] and *Sox10* (*Sox10^flox^*) [14] alleles with a tamoxifen-inducible CreERT2 recombinase under the control of *Plp1* regulatory sequences (*Plp1-CreERT2*) [11]. Mice homozygous for the floxed alleles that lacked *Plp1-CreERT2* served as controls. Genotyping was performed as described previously [11,14,24]. The mice were kept under standard housing conditions with 12:12 h light–dark cycles and continuous access to food and water in accordance with animal welfare laws and ethical regulations. To induce the Cre recombinase, mice were injected with 200 µL of a 10 mg/mL tamoxifen solution on five consecutive days starting on postnatal day (P) 60. Tissue was collected 21 days post induction (21 dpi) from the adult mice.

Animals used for histological stainings and electron microscopy were sacrificed by exposure to carbon dioxide before perfusion with 4% paraformaldehyde (for histology) or 4% paraformaldehyde, 2.5% glutaraldehyde (for electron microscopy), and the dissection of brains. For immunohistochemistry and in situ hybridization, tissues underwent additional overnight fixation in 4% paraformaldehyde followed by extensive washing with phosphate buffered saline (PBS) and cryoprotection in 30% sucrose. Embedding and freezing at −80 °C were performed before cryotome sectioning. Both males and females were used.

The mice used for RNA preparation were anaesthetized with isoflurane and then sacrificed by cervical dislocation. The study was approved by responsible local committees and government bodies (University Erlangen-Nürnberg, Veterinäramt Stadt Erlangen TS-2/2021 Bioch. und Pathobioch. and Regierung von Unterfranken, RUF-55.2.2-2532-2-1735).

### 4.2. Tissue Stainings

The immunohistochemistry on 10 µm-thick sections was performed on slides using the following primary antibodies: rabbit anti-Bcas1 (Synaptic System, Göttingen, Germany, #445-003, 1:1000 dilution); rabbit anti-Gfap (Neo Markers, Portsmouth, NH, USA, #RB-087-A, 1:300 dilution); rabbit anti-Iba1 (WAKO, Richmond, VA, USA, #019-19741, 1:250 dilution); rabbit anti-Plp1 (Abcam, Cambridge, UK, #AB28486, 1:500 dilution); rabbit anti-Sox9 (made in house, 1:1000 dilution) [17]; rabbit anti-Olig2 (Millipore, Billerica, MA, USA, #AB9610, 1:1000 dilution); mouse anti-Olig2 (Millipore, #MABN50, 1:500 dilution); goat anti-Aspa (US Biological, Salem, MA, USA, #L3051757, 1:1000 dilution); goat anti-Mog (Abcam, #AB115597, 1:500 dilution); goat anti-Pdgfra (R&D Systems, Minneapolis, MN, USA, #AF1062, 1:50 dilution); goat anti-Sox10 (made in house, 1:1000 dilution) [32]; guinea pig anti-Sox10 (made in house, 1:1000 dilution) [10]; guinea pig anti-Sox6 (made in house, 1:1000 dilution) [31]; guinea pig anti-Sox8 (made in house, 1:1000 dilution) [10]; and rat anti-Mbp (Serotec, Neuried, Germany, #MCA409S, 1:500 dilution). Secondary antibodies were coupled to Cy3 or Cy5 (Dianova, Hamburg, Germany) fluorescent dyes. Nuclei were counterstained with 4,6-diamidine-2-phenylindole (DAPI). For in situ hybridization, 10 µm-thick cryotome sections were incubated with digoxigenin-labeled antisense riboprobes specific for *Plp1* and *Mog*, as described in [10]. Stainings were analyzed and documented with a DMI6000 B inverted microscope equipped with a DFC 360FX camera (Leica, Wetzlar, Germany).

### 4.3. Electron Microscopy

After perfusion of the mice at 21 dpi (81 days) with a 4% paraformaldehyde and 2.5% glutaraldehyde solution, the brains were dissected and sagittally cut. Sample processing followed established protocols and included post-fixation in 2% osmium tetroxide, dehydration in ascending steps of ethanol and acetone, and embedding in epon [14]. After trimming, as well as ultrathin sectioning and staining with uranyl-acetate and lead citrate, images were acquired at a nominal magnification, as indicated in the figure legend, with a LEO 906E transmission electron microscope (Carl Zeiss Microscopy, Oberkochen, Germany) at 60 kV acceleration voltage.

### 4.4. RNA Sequencing

For RNA sequencing, the corpus callosum was dissected from 4 controls and 4 tamoxifen-injected knockout animals per strain on dpi21. The mice were sacrificed, and their brains were collected, briefly rinsed in cold PBS, and frozen on dry ice. Using a razor blade, the cerebellum was removed, and the remaining frozen sample was cut coronally into five even sections. From Sections 2 to 4, the corpus callosum was dissected under a stereomicroscope. After collection of the tissue, samples were dissociated using a gentleMACS Dissociator, and the total RNA was extracted by a Trizol-based protocol. Contaminating DNA was removed by treatment with DNAse I before determining the quantity, purity, and quality on an Agilent 5300 Fragment Analyzer (Agilent Technologies, Santa Clara, CA, USA). Between 1 µg and 2 µg total RNA per sample were used for library preparation using the NEBNext Ultra II RNA Library Prep Kit for Illumina (NEB, Ipswich, MA, USA) in accordance with the manufacturer’s instructions. The libraries underwent paired-end sequencing, generating an average of 50 million reads per library. Mapping onto the mouse genome mm10 was conducted with STAR aligner (v2.5.2b), and the unique gene hit counts were calculated by using feature counts from the Subread package v1.5.2. DESeq2 was employed for statistical analysis. Data were deposited in GEO and are accessible under GSE269122.

The GO analysis of upregulated and downregulated genes was performed using the Gene Ontology enrichment, analysis, and visualization tool GOrilla [33]. Gene signatures with alterations consequent to *Sox8/Sox10* gene inactivation were identified using the Gene Set Enrichment Analysis (GSEA) tool from the Broad Institute (Cambride, MA, USA) [34]. To characterize the state of oligodendrocytes, the transcript levels of genes coding for myelin proteins (*Mbp, Plp, Mog, Mag, Mal,* and *Opalin*), those associated with the mature state (*Aspa* and *Enpp6*), or those coding for lipid metabolic enzymes (*Fa2h, Elovl1, Enpp6, Gal3st,* and *Ugt8a*) were consulted.

### 4.5. Quantitative RT-PCR (qRT-PCR)

RNA was prepared as described above. After the reverse transcription to cDNA with an oligo-dT primer and Moloney Murine Leukemia Virus reverse transcriptase (NEB), quantitative PCR was performed using a PowerUp SYBR Green Mastermix (Thermo Fisher Scientific, Waltham, MA, USA, A25743) with the following primers: *Fa2h* (5′-TCTGTCTCCTCTCTCCCTGC-3′ and 5′-CCCTTCTTGGCTTCAGGAGG-3′); *Gapdh* (5′-CTTGCTCAGTGTCCTTGCTG-3′ and 5′-CCCACTCTTCCACCTTCGAT-3′); *Mbp* (5′-CCAAGTTCACCCCTACTCCA-3′ and 5′-TAAGTCCCCGTTTCCTGTTG-3′); *Mog* (5′-AGGCCTTGTATTCCTCTTCCTGC-3′ and 5′-GCTCCAGGAAGACACAACC ATCA-3′); *Plp1* (5′-GCTCCTGGTGTTTGCCTGCTC-3′ and 5′-CACCCACAAACGCAGCAATAAA-3′); *Sox8* (5′-ACCCGCATCTCCATAACGCA-3′ and 5′-TGGTGGCCCAGTTCAGTACC-3′); and *Sox10* (5′-ACAGCAGCAGGAAGGCTT CT-3′ and 5′-TGTCCTCAGTGCGTCCTTAG-3′). All samples were processed as technical triplicates. Transcript levels were normalized to *Gapdh* and the data were analyzed by the ΔΔCt method.

### 4.6. Quantifications and Statistical Analysis

The results from independent animals, experiments, or separately generated samples were treated as biological replicates (n ≥ 4). Quantifications of signal intensities and cell numbers in the immunohistochemical stainings and in situ hybridizations, as well as the g-ratio determination (defined as the ratio of the outer diameter of the myelin sheath to the axon diameter) and quantification of unmyelinated axons, were performed using Fiji ImageJ software (version 2.1.0/1.53o). For the immunohistochemical stainings and in situ hybridizations, the corpus callosum of each animal was analyzed on three separate sections. On each section, quantification was performed in three areas of the corpus callosum (one near the midline and one near each lateral edge). For determination of the g-ratio and the number of unmyelinated axons, three electron microscopic images per animal were analyzed. To determine whether the differences in cell numbers were statistically significant, a two-tailed Student’s *t*-test was performed using GraphPad Prism software version 8 (*, *p* ≤ 0.05; **, *p* ≤ 0.01, ***, *p* ≤ 0.001).

## 5. Conclusions

Despite certain limitations, our current study confirms beyond doubt the involvement of both Sox8 and Sox10 in the expression of myelin-related genes and myelin maintenance, as well as the unequal contribution of these two paralogs. Additionally, it provides evidence that the relationship between both proteins and their molecular mode of action is much more complex than expected. Most results can be explained by a model in which Sox8 and Sox10 are present in adult oligodendrocytes in non-limiting amounts and perform very similar functions but with different efficiencies, possibly due to different interactions or interaction efficiencies with other transcription factors, transcriptional co-regulators, or regulatory chromatin molecules. As a consequence, a loss of Sox8 will have no effect on the expression of genes jointly targeted by Sox8 or Sox10, and it might even lead to a slightly increased expression. In contrast, a loss of Sox10 will result in minor reductions in the expression of joint target genes, with dramatic changes only visible upon loss of both Sox8 and Sox10. However, further analysis in future investigations will be required to validate this model. Additionally, we would like to point out that SOX8 is a known genetic risk locus for multiple sclerosis [35]. Whether and how this relates to our findings remains to be elucidated.

## Data Availability

All the data generated or analyzed during this study are included in this published article or were deposited in GEO under accession number GSE269122 [https://www.ncbi.nlm.nih.gov/geo/query/acc.cgi?acc=GSE269122] (accessed on 5 August 2024).
